# Novel protein kinase C phosphorylated kinase inhibitor-matrine suppresses replication of hepatitis B virus via modulating the mitogen-activated protein kinase signal

**DOI:** 10.1080/21655979.2021.2024957

**Published:** 2022-01-17

**Authors:** Shen Zhou, Yuan Li, Jing Gao, Yanyan Wang, Xinping Ma, Hui Ding, Xiuling Li, Suofeng Sun

**Affiliations:** a Department of Gastroenterology, Zhengzhou University People’s Hospital, Henan Provincial People’s Hospital, Zhengzhou, Henan, China; b Department of Traditional Chinese Medicine, The Third Affiliated Hospital Affiliated of Henan University of Traditional Chinese Medicine, Zhengzhou, Henan, China

**Keywords:** Matrine, HBV, PKC, phosphoproteome analysis, MAPK signaling pathway

## Abstract

HBV (hepatitis B virus) infection still threatens human health. Therefore, it is essential to find new effective anti-HBV compounds. Here, we identified matrine as a novel inhibitor of PKC (protein kinase C) phosphorylated kinase by screening a natural compound library. After HepG2.215 cells were treated with matrine, we carried out a phosphorylated proteomics sequence study and analyzed the prediction of related kinase expression level. In the case of HBV infection, it was found that PKC kinase mediates the activation of mitogen-activated protein kinase (MAPK) signaling pathway known as son of sevenless (SOS) activation. It was also found that PKC kinase inhibits the expression of C-X-C Motif Chemokine Ligand 8 (CXCL8) by inhibiting the activity of activating transcription factor 2/ cAMP response element binding protein (ATF2/CREB), and this effect is independent of its activated MAPK signaling pathway. Finally, Western blot was used to detect the expression of MAPK, ATF2, CREB3 phosphorylation and nonphosphorylation in matrine-treated cells and PKC-treated cells. PKC phosphorylated kinase inhibitor-matrine suppresses the replication of HBV via modulating the MAPK/ATF2 signal. Matrine is a good clinical drug to enhance the autoimmunity in the adjuvant treatment of chronic HBV infection.

## Graphical Abstract

**Figure:**
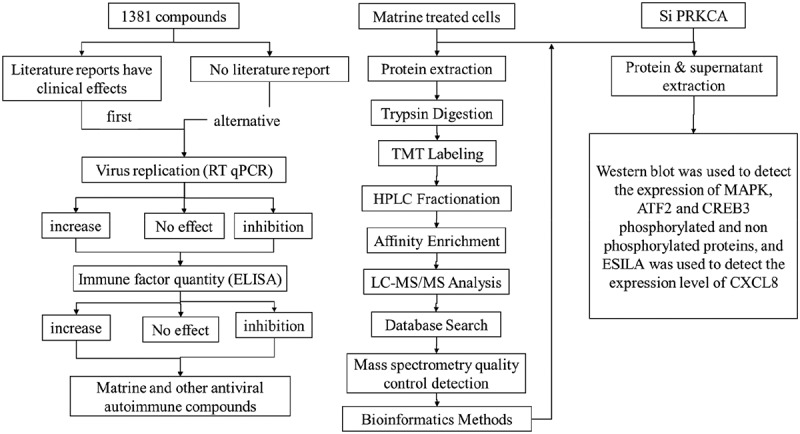

## 1. Introduction

Hepatitis B virus (HBV) infection can cause chronic or acute HBV infection, liver cirrhosis, and hepatocellular carcinoma [1,2]. Even in patients with chronic HBV infection whose laboratory and examination indexes are normal, liver cancer was still found in a review [2,3]. Therefore, the treatment of HBV patients and carriers is still a serious concern in the future [3–5].

Some Chinese herbal medicines, including Chinese herbal formulas, single herbs, and their active ingredients, are frequently reported to have antiviral effects in basic or clinical studies [6,7]. Patients with chronic HBV infection can benefit from traditional Chinese medicine (TCM) treatment [8]. Matrine is the main active component of *Sophora flavescent* against HBV. Matrine exhibits many biological activities such as anti-inflammatory, antivirus, antifibrosis, antiarrhythmia, and immunosuppression activities, leading to wide clinical use in the treatment of HBV infection and liver fibrosis in China [7–10]. A decrease in the HBV DNA level was also observed in cell experiments; however, its mechanism is unclear [9,11–13]. At present, it is not known why matrine can inhibit HBV replication, but it seems that matrine is related to the immune response [14–17].

Cytokine production is a host immune response to viral infection and plays an important role in immune regulation. It can directly inhibit virus replication or achieve antiviral effects by activating innate or adaptive immunity [18–20]. Interleukin-8 is a type of cytokine belonging to the chemokine family, which can mobilize other cells with immune function to achieve the antiviral effect [18,21,22]. HBV reduces the interferon (IFN) level by blocking the recruitment of natural killer cells-γ, secretion of tumor necrosis factor (TNF), and CXCL8 escape natural immunity [23].

The protein kinase C (PKC) family of serine/threonine protein kinases, comprising the ‘classical’ PKC (cPKC), ‘novel’ PKC (nPKC), ‘atypical’ PKC (aPKC), and Protein kinase N (PKN) subfamily, is one of the defining families of the AGC kinase class1 [24–26]. As one of the classical mitogen-activated protein kinase (MAPK) signaling pathways, extracellular signal-regulated kinase (ERK) may be activated by Ca^2+^, PKC, and growth factors to regulate cellular activities. CXCL8 signaling is related to the activation of classic MAPK signaling cascade, with the downstream phosphorylation of ERK-1/2 cancer cells [27–31].

This is the first study on elucidating the mechanism of how matrine can act against HBV. We hypothesized that matrine might assist with immune regulation and anti-inflammatory response, and the body’s immunity is a key factor in controlling viral infection and replication. This study aimed to develop a method to elucidate the action mechanism of various TCMs. Therefore, we can report better compatibility of TCMs by elucidating the molecular mechanism, thus avoiding the waste of resources and money.

## 2. Materials and methods

### 2.1 Cell culture

Human hepatoma cell line HepG2.2.15 was purchased from a typical culture collection center in China. Frozen HepG2.2.15 cells were thawed rapidly at 37°C, centrifuged to discard the frozen solution, added to an MEM medium supplemented with 10% FBS and 25 units/mL penicillin and streptomycin, resuspended and transferred to a culture dish, and the cells were cultured in a 5% CO_2_ incubator at 37°C. HepG2.2.15 cells in 24-well plates were cultured with 300 μL of matrine MEM medium or ordinary MEM medium according to groups. After three days, the medium was changed, and 200 μL of supernatant and cells were collected on the 7th day of the experiment [32].

### 2.2 Preparation of medicated medium

Matrine (Cat. No. HY-N0164) was purchased from MedChem Express. The initial concentration of matrine solution was 10 mM. Matrine was added to the MEM medium in a ratio of 1:10,000, 1:2000, 1:1000, and 1:500 to prepare 1 μM, 5 μM, 10 μM, and 20 μM medicated medium [33–36].

### 2.3 PCR

An HBV nucleic acid assay kit (PCR fluorescent probe method) was purchased from the Da’an gene of Sun Yat-Sen University. First, 200 μL of supernatant sample or HBV positive quantitative reference was placed in a 1000 μL centrifuge tube. Then, 4 μL internal standard solution and 450 μl DNA extract were added to the centrifuge tube and vortexed for 15 s. The mixture was incubated at 100°C for 10 min in a hot water bath prior to centrifugation at 12,000 rpm for 5 min. The reaction mixture (PCR reaction solution and Taq enzyme) and 20 μl of sample were placed in the PCR tube and centrifuged at 8000 rpm for a few seconds.

The cycle conditions were as follows:

93°C for 2 min

93°C for 45 s → 55°C for 60 s → 10 cycles

93°C for 30 s → 55°C for 45 s → 30 cycles [37,38].

### 2.4 Quantitative omics analysis of TMT phosphorylation modification

Through the TMT labeling and phosphorylation modification enrichment technology and the quantitative proteomics research strategy of high-resolution liquid chromatography-mass spectrometry, we carried out a quantitative study of phosphorylation-modified proteomics in this study [39–42]. In this study, 14,328 phosphorylation modification sites were identified on 3826 proteins; among them, 12,377 sites of 3617 proteins contained quantitative information. To ensure a high credibility of the results, we filtered the identification data using localization probability >0.75 and finally determined 11,389 phosphorylation modification sites on 3587 proteins. Among them, 10,707 sites of 3447 proteins contain quantitative information. The screening of differential loci followed the following criteria: 1.2 times as the change threshold, CV < 0.1. Based on the above data and standards, we found that the modification level of 750 sites was upregulated, and 1163 sites were downregulated in the con comparison group. Subsequently, we performed a systematic bioinformatics analysis of proteins containing quantitative information sites, including protein annotation, functional classification, functional enrichment, and cluster analysis based on functional enrichment.

#### 2.4.1 Protein extraction

The sample was sonicated three times on ice using a high intensity ultrasonic processor (Scientz) in lysis buffer (8 M urea, 1% protease inhibitor cocktail). (Note: For PTM experiments, inhibitors were also added to the lysis buffer, eg., 3 μM TSA and 50 mM NAM for acetylation.) The remaining debris was removed by centrifugation at 12,000 g at 4°C for 10 min. Finally, the supernatant was collected, and the protein concentration was determined using a BCA kit according to the manufacturer’s instructions.

#### 2.4.2 Trypsin digestion

For digestion, the protein solution was reduced with 5 mM dithiothreitol for 30 min at 56°C and alkylated with 11 mM iodo acetamide for 15 min at room temperature in darkness. The protein sample was then diluted by adding 100 mM TEAB to urea with a concentration of less than 2 M. Finally, trypsin was added at 1:50 trypsin-to-protein mass ratio for the first digestion overnight and 1:100 trypsin-to-protein mass ratio for a second 4 h digestion.

#### 2.4.3 TMT labeling

After trypsin digestion, the peptide was desalted using a Strata X C18 SPE column (Phenomenex) and vacuum dried. The peptide was reconstituted into 0.5 M TEAB and processed according to the manufacturer’s protocol for TMT kit [41]. Briefly, one unit of TMT reagent was thawed and reconstituted in acetonitrile. The peptide mixtures were then incubated at room temperature for 2 h and pooled, desalted, and dried by vacuum centrifugation.

#### 2.4.4 HPLC fractionation

The tryptic peptides were fractionated into fractions using a high pH reverse-phase HPLC equipped with a Thermo Betasil C18 column (5 μm particles, 10 mm ID, 250 mm length) [43–45]. Briefly, peptides were first separated with a gradient of 8% to 32% acetonitrile (pH 9.0) over 60 min into 60 fractions. Then, the peptides were combined into six fractions and dried by vacuum centrifugation.

#### 2.4.5 Affinity enrichment

Pan antibody based PTM enrichment [46,47]:

To enrich the modified peptides, tryptic peptides dissolved in NETN buffer (100 mM NaCl, 1 mM EDTA, 50 mM Tris-HCl, 0.5% NP-40, pH 8.0) were incubated with prewashed antibody beads (Lot number 001, PTM Bio) at 4°C overnight with gentle shaking. Then, the beads were washed four times with NETN buffer and twice with H_2_O. The bound peptides were eluted from the beads with 0.1% trifluoroacetic acid. Finally, the eluted fractions were combined and vacuum dried. For LC-MS/MS analysis, the resulting peptides were desalted using a C18 ZipTips (Millipore) according to the manufacturer’s instructions.

Biomaterial-based PTM enrichment (for phosphorylation)

The peptide mixtures were first incubated with an IMAC microsphere suspension under vibration in a loading buffer (50% acetonitrile/6% trifluoroacetic acid). The IMAC microspheres with enriched phosphopeptides were collected by centrifugation, and the supernatant was removed [48,49]. To remove nonspecifically adsorbed peptides, the IMAC microspheres were sequentially washed with 50% acetonitrile/6% trifluoroacetic acid and 30% acetonitrile/0.1% trifluoroacetic acid. To elute the enriched phosphopeptides from the IMAC microspheres, an elution buffer containing 10% NH_4_OH was added, and the enriched phosphopeptides were eluted with vibration [39]. The supernatant containing phosphopeptides was collected and lyophilized for LC-MS/MS analysis [50,51].

#### 2.4.6 LC-MS/MS analysis

The tryptic peptides were dissolved in 0.1% formic acid (solvent A) and directly loaded onto a home-made reverse-phase analytical column (15 cm in length, 75 μm i.d.). The gradient was as follows: 6% to 23% solvent B (0.1% formic acid in 98% acetonitrile) over 26 min, 23% to 35% in 8 min, and 80% in 3 min, hold at 80% for the last 3 min, all at a constant flow rate of 400 nL/min in an EASY-nLC 1000 UPLC system.

The peptides were subjected to an NSI source followed by tandem mass spectrometry (MS/MS) in a Q ExactiveTM Plus (Thermo) instrument coupled online to the UPLC. The applied electrospray voltage was 2.0 kV. The m/z scan range was 350 to 1800 for the full scan, and intact peptides were detected in the Orbitrap at a resolution of 70,000. Peptides were then selected for MS/MS using NCE setting as 28, and the fragments were detected in the Orbitrap at a resolution of 17,500. A data-dependent procedure was followed that alternated between one MS scan followed by 20 MS/MS scans with 15.0 s dynamic exclusions. Automatic gain control (AGC) was set at 5E4. Fixed first mass was set as 100 m/z [52].

#### 2.4.7 Database search

The resulting MS/MS data were processed using Maxquant search engine (v.1.5.2.8). Tandem mass spectra were searched against human UniProt database concatenated with the reverse decoy database. Trypsin/P was specified as cleavage enzyme, allowing up to four missing cleavages. The mass tolerance for precursor ions was set at 20 ppm in the first search and 5 ppm in the main search, and the mass tolerance for fragment ions was set as 0.02 Da. Carbamidomethyl on Cys was specified as a fixed modification; acetylation modification and oxidation on Met were specified as variable modifications. FDR was adjusted to <1%, and the minimum scores for modified peptides was set as >40.

### 2.5 Bioinformatics methods

#### 2.5.1 Analysis software

**Figure:**
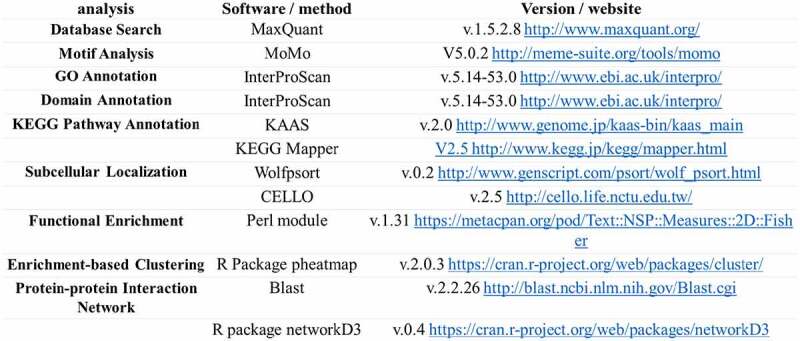

#### 2.5.2 Protein–protein interaction network

All differentially expressed modified protein database accessions or sequences were searched against the STRING database version 10.1 for protein–protein interactions [53]. Only interactions between the proteins belonging to the searched data set were selected, thereby excluding the external candidates. STRING defines a metric called ‘confidence score’ to define interaction confidence; we fetched all interactions that had a confidence score ≥0.7 (high confidence). Interaction network form STRING was visualized in R package ‘networkD3.’

### 2.6 Western blot

For Western blot, a whole cell extract was prepared by lysing cells in NP-40 lysis buffer. Then, the proteins were resolved by 10% SDS-PAGE gel and transferred onto the PVDF membrane. After incubating with the corresponding primary and secondary antibodies, the membrane was washed using TBST, and chemiluminescent signals were developed using Clarity™ Western ECL Substrate (Bio-Rad, CA, USA).

### 2.7 ELISA

For the determination of cell-associated IL-8, the cells were lysed using 1% (v/v) Triton X-100 in PBS prior to the analysis. The concentration of IL-8 was assayed using a commercially available ELISA kit (PeliKine kit; CLB, Amsterdam, The Netherlands) following the manufacturer’s instructions [54]. A double-antibody sandwich immunoassay was carried out using a monoclonal antihuman IL-8 antibody and a biotinylated sheep antibody to human IL-8. A standard curve was obtained using known amounts of natural human IL-8 in the dilution buffer as recommended by the supplier. All the test samples were diluted at least 1:2 in working-strength dilution buffers. Each measurement was performed in duplicate, and the means were used for further analysis. The sensitivity of ELISA was 1–3 pg/mL, and according to the manufacturer, the IL-8 values in serum and plasma of healthy individuals were below 10 pg/mL. Serum and blood cells were separated within 4 h; hemolyzed or lipemic specimens were excluded.

### 2.8 Statistical analysis

Quantitative analyses were performed using SPSS 17.0, and the data were expressed as mean ± s.e.m. Comparisons among groups were performed using Student’s t-test, and other data were analyzed using a one-way analysis of variance (ANOVA).

## 3. Results

We identified Matrine as a novel inhibitor of the PKC (protein kinase C) phosphorylated kinase through the natural compound library screening. After Matrine treated HepG2.215 cells, we carried out phosphorylated proteomics sequence and analyzed prediction of related kinase expression level. Finally, Western blot was used to detect the expression of MAPK, ATF2, CREB3 phosphorylation and non-phosphorylation in Matrine treated cells and PKC a kinase inhibitor treated cells.

### 3.1 Matrine inhibits HBV DNA replication in HepG2.2.15 cells

**Figure 1.**
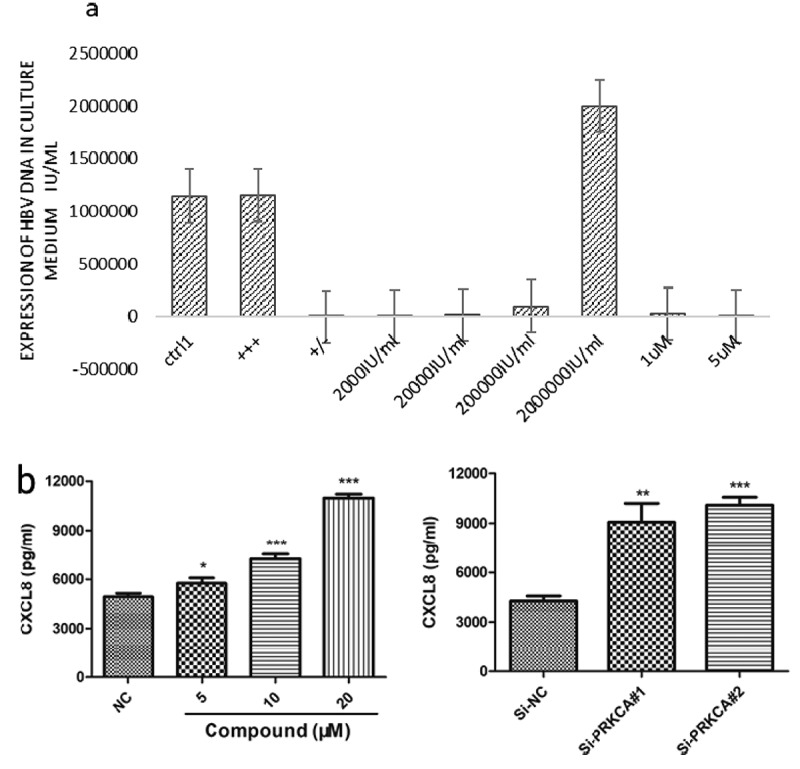
Matrine inhibits HBV DNA replication in HepG2.2.15 cells. (a) HepG2.2.15 cells in 24-well plates were cultured with 300 μL of 1 μM or 5 μM matrine MEM medium or ordinary MEM medium according to groups. After three days, the medium was changed, and 200 μL of supernatant was collected on the 7th day of the experiment. The quantity of HBV DNA in the samples was detected using a PCR fluorescence probe method. (b) The expression of CXCL8 was detected by ELISA.

The virus was strongly positive in the supernatant of the control group, and the serum HBV DNA load was >1 × 10^6 IU/mL. When the concentration of matrine was 1 μM and 5 μM, the level of HBV DNA in the supernatant was 2 * 10 ^ 4 IU/mL and 2 * 10 ^ 3 IU/mL, respectively. The amount of HBV in HBV-positive quantitative reference was expressed in the corresponding order of magnitude, and the measurement error was less than 2 times. No magnitude error change was observed. After matrine treatment, the HBV DNA level in supernatant decreased in a dose-dependent manner (Figure 1(a)). Similarly, we used ELISA to detect the expression level of inflammatory chemokine CXCL8 after treating with matrine at a concentration of 5 μM, 10 μM, and 20 μM. When the cells were treated with 5 μM medicated medium, the expression of inflammatory chemokine CXCL8 increased by 5% compared with the control group (Figure 1(b)). Based on the role of inflammatory chemokine CXCL8 in mobilizing other immune functional cells, we speculate that the antiviral effect of matrine is related to the increase in CXCL8 expression. However, how matrine causes the increase in CXCL8 expression and then enhances the intermediate immunity process is still unclear. We are very interested in the mechanism of its action and analysis of the possible mechanism.

### 3.2 Analysis of phosphorylated proteome in HepG2.215 cells treated with matrine

**Figure 2.**
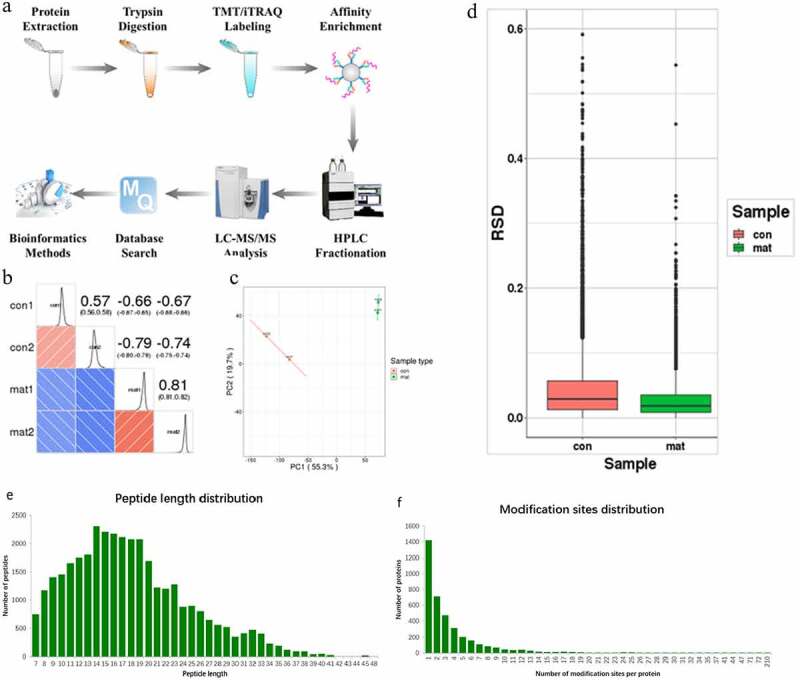
Analysis of the phosphorylated proteome in HepG2.215 cells treated with matrine. (a) Flow chart of sample quantitative phosphorylation proteome identification. HepG2.215 cells were cultured in 5 μM matrine medium and normal MEM medium respectively for 7 days. LC-MS/MS analysis, database search, and bioinformatics analysis were performed as described previously. (b) Pearson correlation coefficient thermogram of quantitative modification between two samples. (c) Two-dimensional scatter plot of PCA distribution of all samples using quantified proteins. (d) Box-plot graphs represent the distribution of sample means calculated for two repeated assays. (e) Peptide length distribution. (f)Distribution of modification sites.

This study further aimed to better understand how matrine enhances CXCL8 expression. According to the technical route shown in Figure 2(a), we systematically quantified the phosphorylated proteome of HepG2.215 cells treated with matrine at a concentration of 5 μM and normal MEM medium. For biologically and technically replicated samples, we examined whether the quantitative results of biological or technical replicates were statistically consistent. Principal component analysis (PCA), relative standard deviation, and Pearson’s correlation coefficient were used to evaluate the quantitative repeatability of proteins. Pearson’s correlation coefficients were as follows.. r = 0.57 (control) and r = 0.81 (experimental group). The Pearson correlation coefficients of the control and experimental groups were – 0.66 to – 0.79. The consistency of the samples satisfied the requirements. A negative correlation was observed between the experimental and control groups (Figure 2(b–d)). The quality control report indicated that this test was consistent with the standards (Figure 2(e-f)). It can be inferred that the inhibition of HBV replication induced by matrine is probably related to the differential expression of some proteins, and our further study was to find these differentially expressed proteins and speculate the activity mode of these proteins.

### 3.3 Matrine-induced differential phosphorylation of nucleoprotein

**Figure 3.**
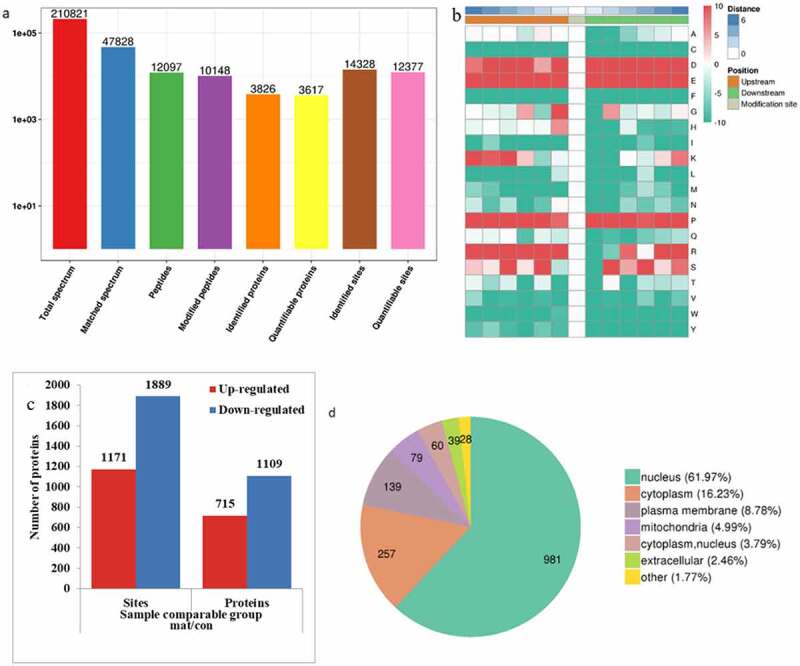
Matrine-induced differential phosphorylation of nucleoprotein. (a) The results of mass spectrum data are provided as a statistical chart. (b) Motif enrichment heat map of phosphorylation. Red color indicates that this amino acid was significantly enriched near the modification site, and green color indicates that the amount of this amino acid was significantly reduced near the modification site. (c) Histogram of differential modified protein and modified site number distribution: The quantitative value of modified peptides corresponding to each sample was detected by mass quantitative spectrometric analysis. For each repeated experiment, the ratio of the quantitative value of modified peptides between two different samples was taken as the differential expression of comparison group (ratio). For each comparison group, the average value of the two repeated ratios was taken as the comparison group’s ratio, and the coefficient of variation, CV of the two repeated ratios as the comparison group’s CV value. When CV value < 0.1, more than 1.2 of the differential expression was taken as the threshold of significant upregulation, and less than 1/1.2 was taken as the threshold of significant downregulation. (d) Subcellular localization of proteins corresponding to different phosphorylation sites.

We quantified and analyzed proteins obtained from the raw database. In this study, 14,328 phosphorylation sites were identified on 3,826 proteins. Among them, 12,377 sites on 3,617 proteins contained quantitative information (Figure 3(a)). A total of 1824 proteins and 2060 phosphorylation sites were detected by mass spectrometry. Among them, 1109 proteins and 1889 phosphorylation sites were significantly downregulated (Figure 3(c)). According to the statistics of amino acid sequences before and after all phosphorylation sites were downregulated in the samples, the trend of amino acid sequences in the phosphorylation sites was calculated. Soft MoMo (motif-x algorithm) was used to analyze the model of sequences constituted with amino acids in specific positions of modify-21-mers (10 amino acids upstream and downstream of the site, but phosphorylation with modify-13-mers i.e., six amino acids upstream and downstream of the site) in all protein sequences. All the database protein sequences were used as background database parameters. The minimum number of occurrences was set to 20. Emulate original motif-x was ticked, and the other parameters were marked as default. Several representative amino acids such as glutamic acid (E), lysine (K) and aspartic acid (D) were enriched downstream of the phosphorylation modification site region. To characterize these differently changed proteins, WoLF PSORT software was used to predict and classify the subcellular localization: 61.97% of the differential phosphorylation sites were located in the nucleus, 16.23% in the cytoplasm, and 8.78% in the cell membrane. The differential modification of nuclear protein might increase the expression of CXCL8 and inhibit HBV replication.

### 3.4 Differential modifications were significantly enriched in some functional types

**Figure 4.**
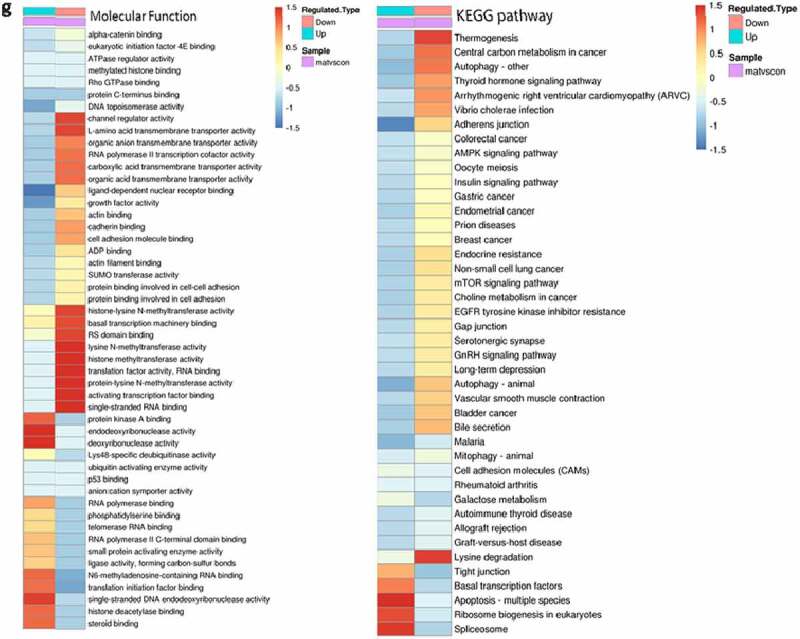
Continued.


As mentioned above, we found that matrine can cause differential phosphorylation of nuclear proteins. Then, to determine the functional classification, the protein functions and classifications were analyzed based on GO, KEGG, and COG, and expressive hierarchical cluster and functional enrichment analyses of differentially expressed proteins were carried out. Among the 25 KOG categories, the cluster of ‘General function prediction’ accounted for the largest proportion (230, 17.3%), followed by ‘Signal transduction mechanisms’ (219, 16.5%) and ‘Transcription’ (132, 10%) (Figure 4(a)). Figure 4(b) shows the top 20 pathways with the most significant protein enrichment corresponding to the differential phosphorylation modification sites. Among them, the proportion of seven types of differential modification proteins in this functional type changes by more than 1.74 times compared with the proportion of identification proteins. Except for the nonsignificant (P > 0.03) enrichment, there are four types of Go classification. The four enrichment pathways are as follows: histone demethylase activity (H3-K9 specific), PKC activity, thyroid hormone receptor binding, and histone-lysine N-methyltransferase activity. In the schematic diagram of KEGG pathway, we found that although the corresponding proteins are present in different KEGG pathways, the differentphosphorylation sites were identical. We speculate that there are several key proteins in matrine-induced differential modification proteins that play a node role.**Figure 4.**
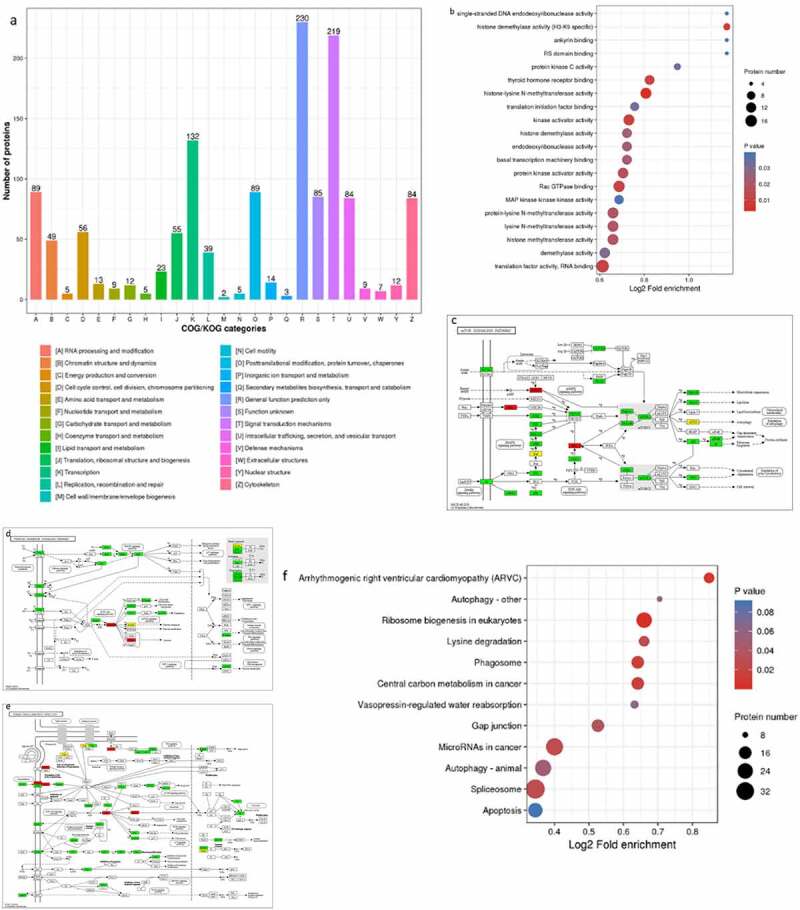
Differential modifications were significantly enriched in some functional types. (a) COG/KOG categories of differential modified proteins. (b) The proteins corresponding to different phosphorylation sites in Molecular Function. The vertical axis shows the functional classification or pathway, and the horizontal axis shows a log2-converted value of the proportion of differential protein in this functional type compared with the multiple of the proportion of identifying proteins. The circle color indicates the enrichment of significant P-value, and the circle size indicates the number of differential proteins in the functional class or pathway. (c–e) Visual display of significant enrichment of proteins corresponding to differential modification sites in a KEGG pathway. Red color indicates differently upregulated proteins; green color indicates differently downregulated proteins; yellow color indicates the presence of multiple proteins in this node, including differently upregulated and differently downregulated proteins. (f) The protein corresponding to different phosphorylation sites was enriched and distributed in KEGG pathway. (g) Cluster analysis heat map. The color blocks, corresponding to the differently expressed proteins and functional descriptions of different groups, indicate the degree of enrichment. Red color indicates strong enrichment, and blue color indicates weak enrichment.

### 3.5 Matrine promotes CXCL8 expression through the PKC-dependent signaling pathway

**Figure 5.**
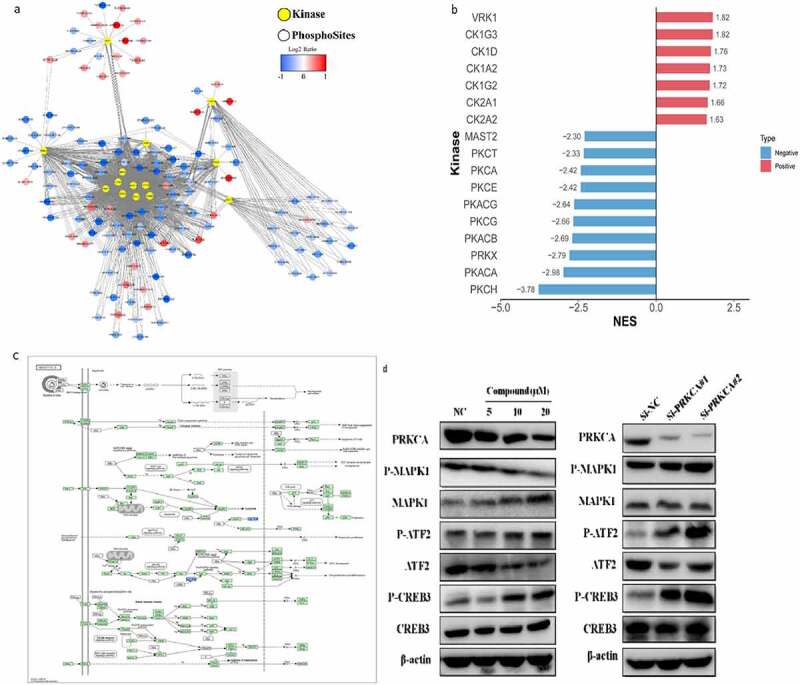
Matrine promotes the expression of CXCL8 through the PKC-dependent signaling pathway. (a) Protein–protein interaction network between kinase and substrate. The yellow octagon represents the kinase, and the circle represents the phosphorylation site. The red circle represents the upregulation site, and the blue circle represent the downregulation site. (b) Highly enriched kinase. (The NOM p-value was less than 0.01) Red was highly upregulated kinase, and blue was highly downregulated kinase. (c) The KEGG pathway. (d) Western blot was used to detect the expression of MAPK, ATF2, and CREB3 phosphorylation and nonphosphorylation in matrine-treated cells and PKC-treated cells.

To find the key point of matrine antiviral effect, we looked for protein kinases that may participate in matrine-induced inhibition of HBV replication. All phosphorylation sites with localization probability >0.75 were analyzed. The differential expression sites and kinase network information were plotted (Figure 5(a)); the significantly enriched kinases were plotted as a histogram (Figure 5(b)). Direct mapping showed that seven upregulated kinases and ten downregulated kinases were induced by matrine (Figure 5(b)). The results show that the kinase activities of PKACA, PKACB, PKACG, PKCA, PKCE, PKCG, PKCH, and PKCT in PKA and PKC families were highly downregulated by matrine. Combined with the signal pathway in human HBV infection shown in Figure 5(c), PKC is an important kinase in the MAPK/ATF2 signaling pathway. According to previous studies, cPKC participates in the regulation of CXCL8. As shown in Figure 4(c–e), matrine inhibits the phosphorylation of multiple kinases in the MAPK signaling pathway. Western blot analysis revealed that an increase in the matrine concentration led to a significant decrease in the expression of PKCa kinase and phosphorylated MAPK1 (Figure 5(d)). Similarly, we studied the expression of its downstream ATF2, CREB3, and the final cytokine CXCL8 and found that matrine can regulate the downstream signal by downregulating the PKC kinase activity. Therefore, matrine regulates MAPK/ATF2 signal pathway through the PKC-dependent signal network, promotes the expression of proinflammatory factor CXCL8, and plays a role in regulating innate immunity.

## 4. Discussion

HBV infection still threatens human health. Therefore, it is essential to find new effective anti-HBV compounds. At present, it is not reported why matrine can inhibit HBV replication, but it seems that matrine is related to the immune response [14–17]. This is the first study on the mechanism of how matrine can act against HBV. We hypothesized that matrine may assist with immune regulation and anti-inflammatory response, and the body’s immunity is a key factor in controlling viral infection and replication. Here, we identified matrine as a novel inhibitor of the PKC phosphorylated kinase by screening a natural compound library. After HepG2.215 cells were treated with matrine, we carried out a phosphorylated proteomics sequence study and analyzed the prediction of related kinase expression level. Finally, Western blot was used to detect the expression of MAPK, ATF2, CREB3 phosphorylation and nonphosphorylation in Matrine-treated cells and PKC-treated cells. Matrine is a good clinical drug to enhance autoimmunity in the adjuvant treatment of chronic HVB infection. This study provides a direction for the simplification and economic improvement of the existing TCM prescriptions.

Despite the widespread use of vaccines and antiviral drugs, 3.5% of the global population is suffering from chronic HBV infection, who carry a high risk of cirrhosis and liver carcinoma [23]. Therefore, it is very important to control the replication of HBV and improve the prognosis of patients with chronic HBV infection.

As one of the components of TCM, *S. flavescent*, matrine has anti-HBV, antiliver fibrosis, anticancer, and immunoregulatory activities [7–9]. Matrine can inhibit the replication of HBV DNA (22–25), and when combined with antiviral drugs, it can enhance the therapeutic effect of antiviral drugs [8,11,13,55]. In this study, we found that matrine could reduce the copy number of HBV DNA in the supernatant from 2 * 10 ^ 6 IU/mL to 2 * 10 ^ 3 IU/mL (Figure 1). Therefore, we confirmed that matrine is a potential candidate drug to control HBV replication and improve its prognosis.

Mass spectrometry is a powerful method to explore the systemic pharmacology [56–59]. We used proteomics and phosphorylation proteomics to reveal the mechanism (Figure 2(a)). Most of the differentially modified proteins were located in nucleoprotein (61.97%). Most of them were signal transduction or transcription proteins (26.5%). By identifying potentially important protein kinases, we found that the PKC family plays the main role (Figure 5(b)). Matrine regulates MAPK/ATF2 signal pathway through the PKC-dependent signal network and promotes the expression of proinflammatory factor CXCL8.

The PKC family of serine/threonine protein kinases, comprising the ‘classical’ PKC (cPKC),‘novel’ PKC (nPKC), ‘atypical’ PKC (aPKC), and PKN subfamilies, is one of the defining families of the AGC kinase class1 [24–26]. In the study of HBx, Schneider R found that HBx induced transactivation through the complex signal transduction pathway, and this activation was partly dependent on the role of PKC [27–31]. PKC kinase activates a number of transcription factors such AP-1/-NF-κ B and CXCL8 by activating the MAPK signaling pathway [30,60]. As an important part of the body’s immune response, cytokines play an important role in the immune response and immune regulation of hepatitis virus infection [18–20]. Interleukin-2 and interleukin-18 exhibited anti-HBV effects in previous studies [61,62]. Interleukin-8 is a type of cytokine belonging to the chemokine family, which can mobilize other cells with immune function to achieve the antiviral effect [18,21,22].

**Figure 6.**
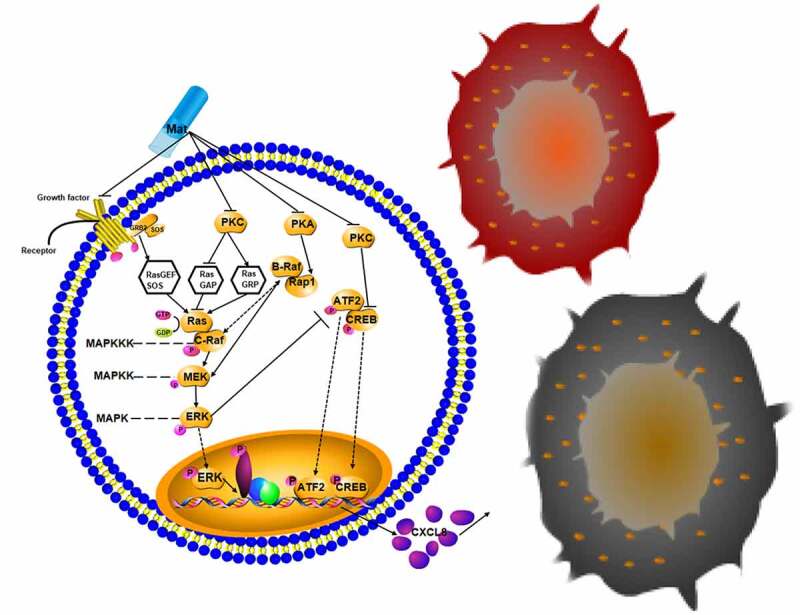
Mechanism showing that matrine increases the expression of inflammatory chemokine CXCL8 to inhibit HBV DNA replication through the PKC-MAPK-ATF2/CREB signaling pathway.

To illustrate our findings more clearly, the molecular mechanism is shown Figure 6. Matrine downregulated the activity of PKC protein kinase family, inactivated MAPK-ATF2/CREB signaling pathway, and then upregulated the expression of CXCL8. In addition, we also found that matrine has more complex regulation of MAPK than PKC protein kinase. When the drug concentration was 20 μM, the expression of PKCa kinase and phosphorylated MAPK1 was less than half of that of the control group (Figure 5(d)). However, PKCa inhibitor was used to inhibit PKCa kinase, we did not find a significant difference in MAPK1 phosphorylation even though PKCa was more significantly inhibited (Figure 5(d)). Combined with the study of Schneider R et al. mentioned above, we speculate that a more complex regulatory network is responsible for the inhibition of the MAPK signaling pathway by matrine. Matrine not only regulates the phosphorylation of the MAPK signaling pathway through PKC kinase but also regulates the upstream signal of RAF through other targets. Whether this inhibitory effect is related to other downregulated kinases such as PKA family or other PKCa family members remains to be further verified (Figure 6).

In conclusion, we developed a method for phosphorylation proteomics analysis combined with the identification of potentially important protein kinases to elucidate the mechanism of drug action. This study confirmed that matrine increase the expression of inflammatory chemokine CXCL8 in inhibiting HBV DNA replication through the PKC-MAPK-ATF2/CREB signaling pathway. This provides a potential therapeutic drug for patients with chronic HBV infection.

## Conclusion

In the case of HBV infection, PKC phosphorylated kinase inhibitor-matrine suppresses the replication of HBV by modulating the MAPK/ATF2 signal. Matrine is a good clinical drug to enhance autoimmunity in the adjuvant treatment of chronic HVB infection. This study was designed to elucidate the mechanism of TCMs, providing a direction for the simplification and economic improvement of the existing TCM prescriptions.

## Limitation of the study and future direction

We know that PKC is closely related to the occurrence and development of HCC. We speculate that the antitumor effect of matrine might also be mediated by PKC kinase, which needs further experiments to prove. In addition, although we have confirmed that matrine can increase the expression of CXCL8 by inhibiting PCA kinase activity, the mode of action of matrine in inhibiting PCA kinase activity is still unknown. However, only from its antiviral activity, it was found that matrine is a good clinical drug to enhance autoimmunity in the adjuvant treatment of chronic HVB infection. Further research on clinical pharmacodynamics, pharmacology, and safety is also essential. We hope that this effect of matrine in enhancing the innate antiviral effect would provide a new treatment for patients with poor response to antiviral drugs or drug resistance.

## Abbreviations

**PKC**: protein kinase C

**PKA**: protein kinase A

**HBV**: hepatitis B virus

**MAPK**: mitogen-activated protein kinase

**SOS**: son of sevenless

**CXCL8**: C-X-C Motif Chemokine Ligand 8

**ATF2**: Activating Transcription Factor **2**

**CREB**: cAMP response element binding protein

**IFN**: Interferon

**TNF**: tumor necrosis factor

**cPKC**: ‘classical’ PKC

**nPKC**: ‘novel’ PKC

**aPKC**: ‘atypical’ PKC

**PKN**: Protein kinase N

**DNA**: deoxyribonucleic acid

**PCR**: Polymerase Chain Reaction

**TMT**: Tandem mass tag

**HPLC**: High Performance Liquid Chromatography

**PTM**: Post-translational modification

**IMAC**: Immobilized metal-ion affinity chromatography

**LC-MS/MS**: Liquid chromatography-tandem mass spectrometry

**ELISA**: enzyme linked immunosorbent assay

**PCA**: Principal component analysis

**MoMo**: motif-x algorithm

**GO**: Gene Ontology

**KEGG**: Kyoto Encyclopedia of Genes and Genomes

**COG**: Cluster of Orthologous Groups of proteins

**KOG**: Clusters of orthologous groups for eukaryotic complete genomes

**AP-1**: activating protein-1

**NF - κ B**: nuclear factor kappa-B

**HBx**: hepatitis B virus X protein
